# Tissue scaffold architecture affects implant degradation and bone tissue regeneration: A novel *in silico* mechanobiological model analysing cell behavior, mechanical stress and degradation kinematics

**DOI:** 10.1371/journal.pone.0349708

**Published:** 2026-05-28

**Authors:** Adel Alshammari, Fahad Alabdah, Lutong Li, Glen Cooper

**Affiliations:** 1 Department of Mechanical and Aerospace Engineering, School of Engineering, University of Manchester, Manchester, United Kingdom; 2 Department of Mechanical Engineering, University of Ha’il, Ha’il, Kingdom of Saudi Arabia; Khalifa University of Science and Technology, UNITED ARAB EMIRATES

## Abstract

Synthetic bone tissue scaffold function is controlled by both material and architecture. Experimental biomaterial approaches have brought significant advances in scaffold function, but scaffold architecture has not been fully explored. There are many scaffold architecture design options which could be more efficiently evaluated using computational methods. The aim of this study is to introduce a novel mechanobiological computational model to assess the effect of implant degradation, bone formation, and the influence of bone loading. A finite element model of a synthetic bone tissue scaffold within a rat bone defect supported by a fixation plate was coupled with an agent-based cell and degradation model (both bulk and surface degradation). The approach was partially validated using *in vivo* experimental mass-loss data and tested in a case study examining four poly-L-lactic acid tissue scaffolds with varying architectures. The model was run for 90 days to calculate results on cell behaviour, tissue formation and scaffold degradation. The results showed that scaffold architecture strongly influences degradation and cellular behaviour, with a filament thickness of 0.6 mm yielding 39 mm³ of new bone formation compared to 18 mm³ in a filament thickness of 0.2 mm, representing an approximate 117% increase at day 90. Cell migration was increased in higher porosity scaffold architectures by 31% when changing from 20.9% (T4) to 54.7% (T1) porosity. The mechanobiological computational model is, to the authors’ knowledge, the first time that implant degradation kinetics, mechanical environment, and cellular behavior have been combined in an *in silico* approach. The results show the importance of scaffold architecture design in the function of bone healing aided by tissue scaffold technology, emphasizing the importance of shape as well as material to improve implant function. Future work should aim to improve degradation modelling to include localised pH, autocatalysis and varying degradation rates due to chemical changes. Additionally, models should also include angiogenesis to account for the importance of revascularization in bone healing.

## 1. Introduction

Globally there are approximately two million large bone fracture cases that occur every year [1,2]. The gold standard for solving these issues is autogenous bone grafts [3–5]. However, there are some drawbacks of this approach such as limited bone tissue availability, disease transmission, a higher rate of infections, and a requirement for secondary surgery.

A jawbone, joint, femur, or any type of bone in the body is not completely solid; it has a porous internal structure. Among other functions, these pores allow the inflow of nutrition, providing good conditions for cells to grow and attach, showing the importance of the natural extracellular matrix microstructure. Following a biomimicry approach, an alternative solution is a synthetic bone graft created using a tissue scaffold approach, which has shown promising results [6] However, these artificial bone tissue scaffolds have some biological and mechanical requirements, such as biocompatibility, biodegradability, and porosity, to allow cell attachment, proliferation, and differentiation [7–10].

**Fig 1:**
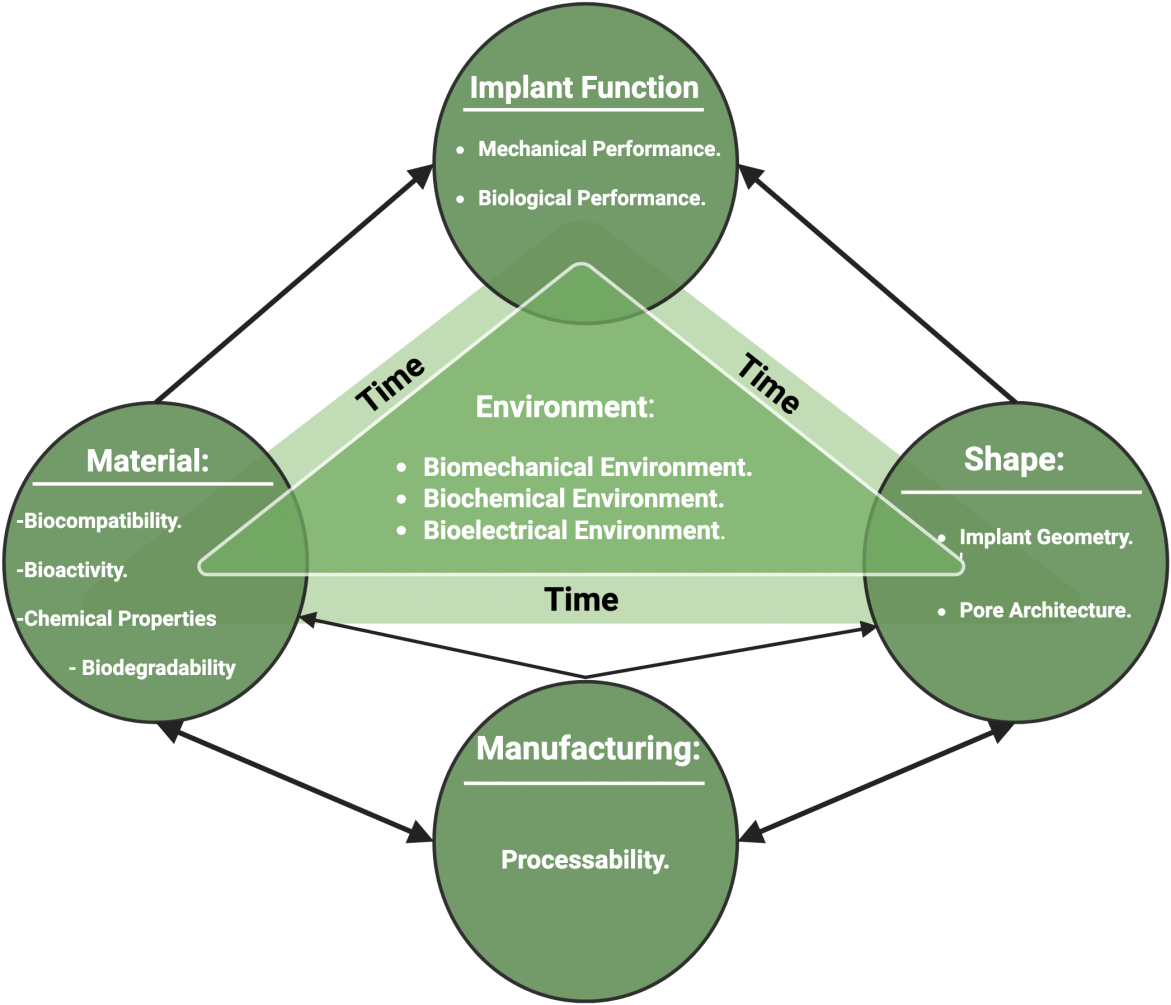
A schematic showing the relationships among material, shape, manufacturing, environmental factors and time influencing implant function.

Both the material and the shape of tissue scaffolds are important to enable biological, mechanical and degradation function [11–16] A lot of valuable research has been conducted on bone graft materials, but less has been outworked on bone graft shape, yet shape is equally important to enable successful bone graft function. This is illustrated in Fig 1, which shows the relationships of function, material, shape, manufacture, and environment, which was first reported in the authors’ previous work [1]. Tissue scaffold shape, specifically pore architecture, is key to providing both mechanical and biological environments for cells. Larger scaffold pores enhance vascularization and cellular infiltration but often compromise mechanical strength, highlighting the need for a balance between biological performance and structural stability [17,18]. Triangular pore shapes showed better mechanical outcomes than circular pore shapes [11,19–21], and round pore shapes are less biologically functional than square pore shapes [13]. The microstructure design of scaffolds affects their functionality in addition to other factors such as the material and the site of fracture. Yet a full understanding of the effect of scaffold architecture on the mechanical and biological aspects of bone healing is still incomplete, meaning that the design space is underexplored, which would require a huge number of experiments.

Other industries use a virtual prototyping method before making physical prototypes, which is different from tissue scaffold research, which focuses on an experimental approach, probably due to the lack of quantified understanding of the mechanobiological processes. However, if an *in silico* mechanobiological approach was available that combined the mechanical and biological aspects, it would lead to a reduced number of experiments, reduced development costs, and quicker development time, but it would require full understanding and quantification of the engineering biology.

For the implementation of a mechanobiological *in silico* model, it is important to understand how mechanical stimulation affects bone cells. Many *in vitro* studies previously showed that mesenchymal stem cells (MSCs) are responsive and sensitive to mechanical stimulations [3,4] which can be modified by both the material and shape of the scaffold. An additional factor that should be taken into consideration in designing bone tissue scaffolds is biodegradation, which affects the mechanical stability through shape change over time, leading to a changing mechanostimulus experienced by cells. This could also affect the biochemical environment, giving further changes in cell behaviour.

Accumulated experimental evidence demonstrates that mechanical cues are potent regulators of cellular activity and matrix organization in bone tissue engineering [22]. *In vitro*, perfusion bioreactors that impose well-defined fluid shear enhance osteoblastic differentiation and mineralized matrix deposition within 3D scaffolds [23], while cyclic mechanical strain elevates osteogenic gene expression and promotes mineralization [24]. Sustained cyclic stretch further aligns osteoblasts and induces anisotropic collagen architecture, linking cell orientation and matrix patterning to the loading direction [25]. Mechanotransduction within bone cells is a key regulator of osteoblast differentiation and the overall process of bone remodeling [26]. Other *in vivo* studies have demonstrated the mechanobiological regulation of healing. In a rat fracture model (no scaffold), Claes and Heigele [27] quantified how local strain and hydrostatic pressure govern tissue fate, fibrous tissue, cartilage, or bone, defining thresholds for mechanoregulated differentiation. Building on this paradigm, Pobloth et al. showed that mechanobiology-informed, stiffness-tuned implants improved load sharing and yielded superior bridging of large segmental defects in sheep [27,28]. Together, these investigations provide a rigorous experimental basis for mechanobiology-coupled models and highlight the need to deliberately engineer the evolving mechanical milieu in construct design.

Previous pioneering research has been done on the effect of the mechanical loading on bone growth, such as shear strain and hydrostatic pressure [29], strain and Perrin’s interfragmentary strain theory [30], octahedral shear strain and flow velocity [22], octahedral shear strain and interstitial flow velocity gradient [31], and strain and hydrostatic pressure [27]. These mechanoregulatory models have been used in many *in silico* approaches [1,32–36]. Previous *in silico* mechanobiological models showed validity and effectiveness in predicting bone growth; however, they are limited to nondegradable scaffolds.

Biodegradation can be very functionally effective in tissue engineering, offering the ability to change the structural support through the healing process that facilitates cell adhesion, proliferation, and differentiation, as well as allowing the implant to be fully replaced with natural tissue in the longer term. Polycaprolactone (PCL) and polylactic acid (PLA) are widely used in tissue engineering due to their cost-effectiveness, biodegradability, and excellent printability, enabling the accurate construction of intricate scaffold architectures designed for regenerative applications [37].

The degradation of scaffolds has been thoroughly experimentally investigated through a variety of *in vitro* and *in vivo* studies. *In vitro* experiments create a controlled environment to examine degradation kinetics and observe alterations in mechanical properties over time, yielding important insights into the potential behaviour of scaffolds in physiological conditions. For instance, studies have been carried out on the degradation patterns of polymer-based thin films and scaffolds to improve their structural design and increase their effectiveness for bone tissue engineering applications [38]. *In vivo* studies, particularly those investigating the degradation of supramolecular polymers in cardiovascular tissue engineering, offer a crucial understanding of scaffold behaviour within living systems. These studies highlight the importance of aligning scaffold degradation rates with the speed of natural tissue regeneration to achieve optimal integration and functionality [39]. Despite the advancements achieved, there remains a gap in computational modelling of biodegradation. Earlier studies have investigated degradation dynamics via experimental *in vitro* and *in vivo* approaches [38,40,41], and others have developed simpler computer models which included bulk degradation [42] and a mathematical diffusion equation [43]. To the authors' knowledge no quantified mechanobiological model exists which includes degradation kinetics, cell behaviour and the mechanical stress environment coupling the impact of mechanical stimuli on tissue formation. Without quantified relationships of biodegradation dynamics, it is impossible to create a comprehensive *in silico* model. Addressing this gap is essential for creating predictive models that can advance scaffold design and increase their efficacy in tissue engineering applications.

This study introduces an innovative computational model that simulates scaffold degradation and bone tissue regeneration by integrating surface and bulk degradation mechanisms within a mechanobiological framework. By proposing equations for degradation dynamics and combining these with mechanobiological concepts, this study aims to develop and validate a novel *in silico* model. The present model dynamically couples time-dependent scaffold degradation with the evolving mechanical and biological environments in order to investigate the effect of scaffold architecture, specifically filament thickness and porosity, on degradation kinetics, cellular behaviors (including MSC migration, proliferation, differentiation and apoptosis), and bone tissue regeneration. This builds on our previous mechanobiological model of the evolving mechanical and biological tissue changes for a non-degradable bone tissue scaffold [1].

## 2. Materials and methods

### 2.1. Mechanobiological model

**Fig 2:**
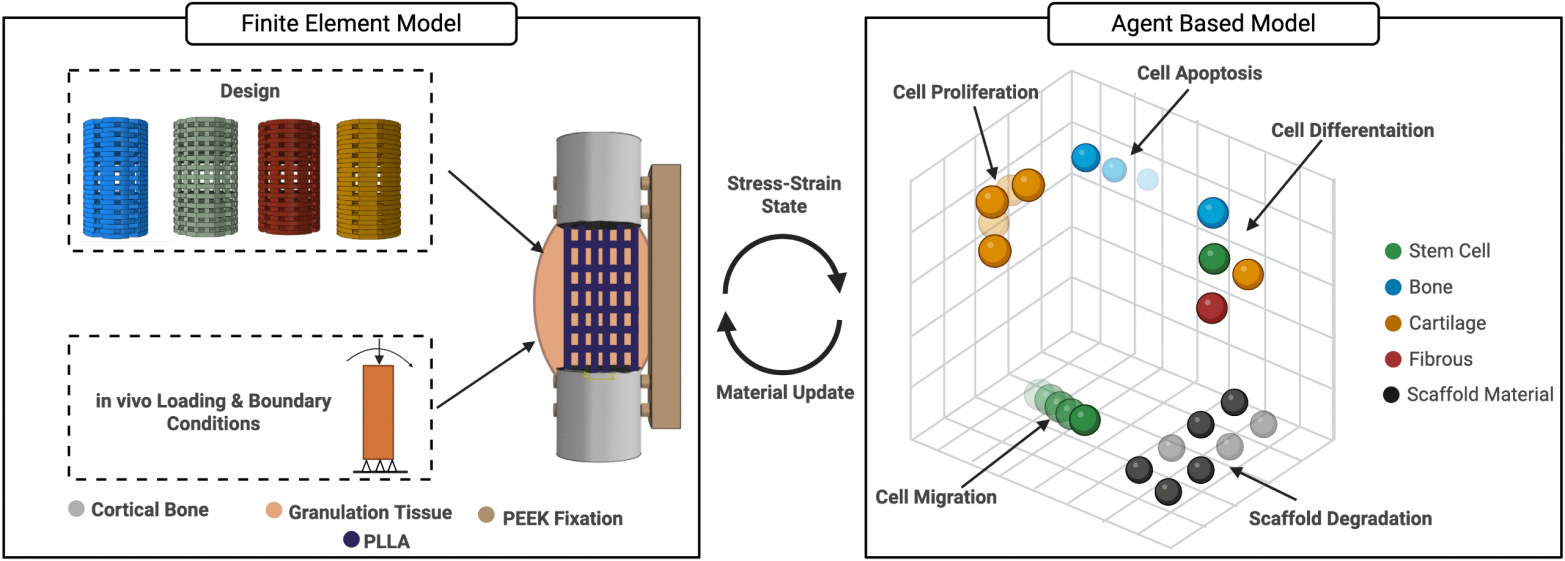
A multi-scale mechanobiological model which couples a finite element model with an agent-based cell behavior-degradation model.

The mechanical stress, scaffold degradation and cell actions to create new tissue are modelled by coupling a finite element model, described in section 2.1.1, with an agent-based cell and degradation model, described in section 2.1.2. The principle of how this model works is illustrated in Fig 2.

#### 2.1.1. Finite element model (FEM).

Previous bone tissue scaffold models use a linear elastic framework to represent the mechanical properties of the scaffold material alongside the surrounding tissue, integrating key parameters like Young’s modulus and Poisson’s ratio to simulate physiological loading scenarios [1,44]. This method allows for the mapping of stress and strain distributions within the scaffold and callus, effectively identifying areas with minimal principal strains and hydrostatic stresses. Expanding upon the foundational research conducted by Claes et al. (1999) [27] pinpointing these high-stress zones is essential for comprehending the influence of mechanical stimuli on cellular behaviour and the promotion of bone formation. This modelling strategy offers a strong and adaptable framework for the design and evaluation of biodegradable scaffolds in the field of regenerative medicine.

**Fig 3:**
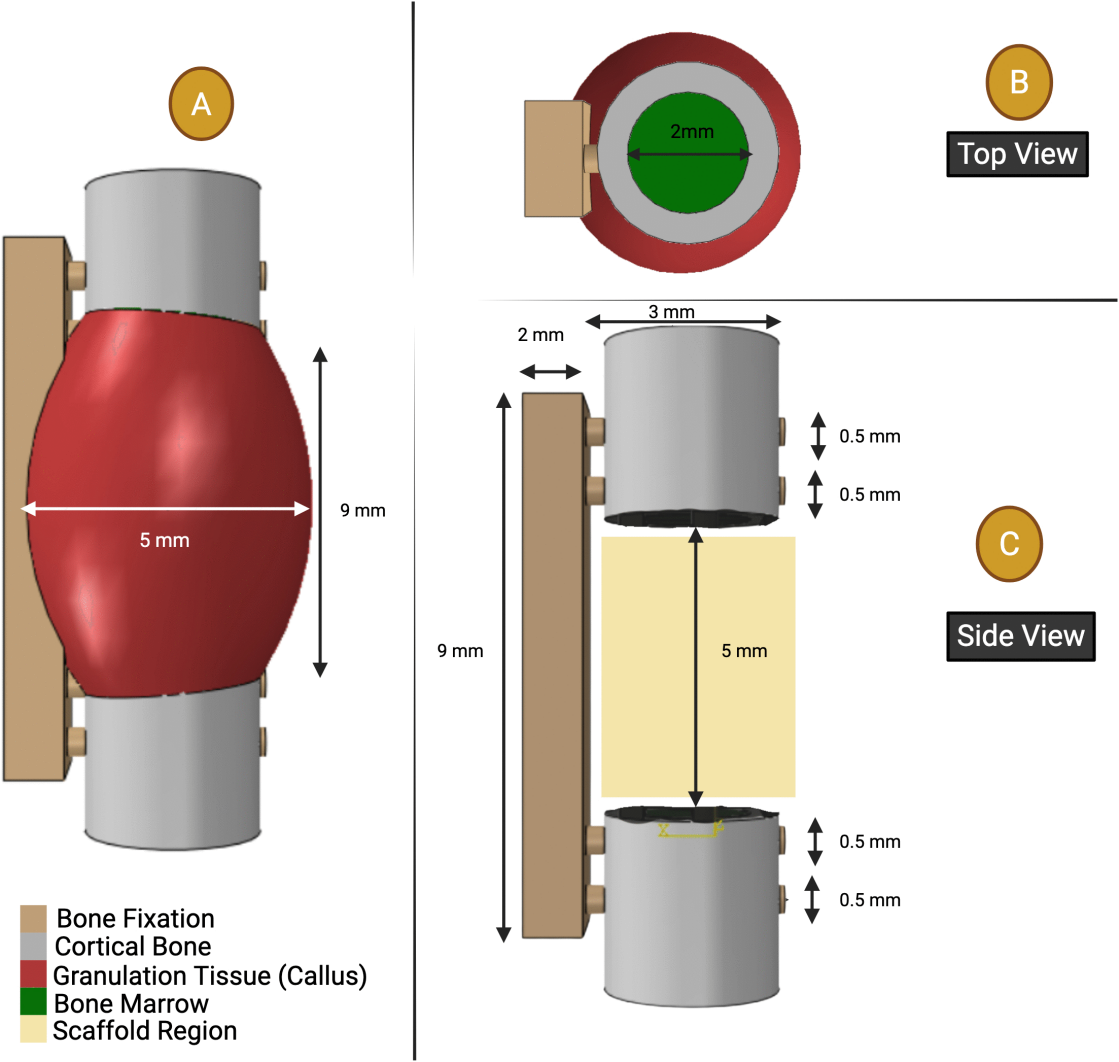
Finite element model geometry: (A) geometry of the callus region. (**B**) dimensions of the bone marrow, and **(C)** cortical bone, bone fixation with four screws, and a 5 mm rat femoral segmental defect regeneration.

Abaqus Standard 2022 (Simulia, Providence, RI, USA) was used to create the model. The geometry had eleven parts: a bone fixation plate (rectangular block 9 mm x 2 mm), secured by four cylindrical screws (0.5 mm diameter, 3.5 mm length), two cortical bones, two bone marrow regions, a scaffold, and a callus section, see Fig 3. The two cortical bones were designed as tubular formations encasing the bone marrow, each with a cortical thickness of 0.5 mm. The bone marrow areas measured 2 mm in diameter and 3 mm in length. An arc-shaped callus structure was integrated to emulate histology representations from experimental research. This arc, measuring 9 mm in length and 5 mm in diameter, overlapped the cortical bone shape and maintained consistency throughout all validation scenarios and design changes. However, the inner volume of the callus was selectively subtracted based on the specific scaffold geometry applied in each case study.

The shape of a bone scaffold implant is customized to individual cases; however, the components of the model are universally applicable across all cases, comprising bone, fixation, and callus parts. The callus was a homogeneous, linear elastic tissue (granulation tissue, amorphous solid) that occupied the pores of the bone scaffold implant during the initial phase of healing and encircled the bone defect. Granulation tissue is assumed also to occupy the empty areas created by the scaffold degradation process, which will be explained in detail in section 2.1.2.

Material properties for the FE model were taken from the literature, as shown in Table 1.

**Table 1:** Material properties used in the computer model [45–49].

Material	Young’s modulus (MPa)	Poisson’s ratio
Granulation tissue	0.2	0.167
Fibrous tissue	2	0.167
Cartilage	10	0.3
Cortical Bone	8,000	0.3
Bone marrow	2	0.167
Poly (l-lactic acid) (PLLA)	1643	0.3
Polyether-ether-ketone fixation (PEEK)	3800	0.36

The model used tetrahedral structural elements (C3D10), to mesh the geometry with a regular mesh size across all the parts. A mesh sensitivity analysis was conducted on the complete defect and scaffold finite element model. Convergence was achieved at a mesh size of 0.35 mm, which was selected as the optimal mesh size for the entire model.

The biomechanical loading was determined from an experimental investigation involving the femur bone of rats during locomotion [50]. A compression load of 17.7 N was applied to the cortical bone on the proximal side [51], representing six times the body weight and simulating peak loading during gait [50]; the weight of the rat, based on experimental data from the literature, was 300 g [52]. A shear load of 5.7 N was applied at the distal end of the bone in the model (mid-shaft of the femoral bone), corresponding to 10.7 times body weight, representing the maximum shear load that generates the highest bending moment during gait [50]. The proximal end of the model, encompassing the cortical bone and bone marrow regions, was restricted in all degrees of freedom through the application of tie constraints. To ensure consistent displacement of the connecting nodes, tie constraints were utilized to secure four bone fixation screws along with their corresponding four holes in the intact bone, which includes both cortical and bone marrow regions.

The callus composition was initially modeled as granulation tissue. After that, the elements of callus were updated every iteration, simulating the actual bone formation by changing the material properties based on the new cell type, following the rule of mixture [53], which was calculated from the agent-based model (Section2.1.2), see Fig 2. Every element’s material properties are modeled based on the averaged mechanical properties of this cell type.

The scaffold was initially modeled as PLLA, see Table 1. After that, elements of the scaffold were updated every iteration based on the agent-based model (degradation model, Section 2.1.2), see Fig 2. The degraded scaffold elements’ mechanical properties change to granulation tissue first and then they change to different cell types based on the agent-based model (Section 2.1.2). This will enable modelling of the scaffold degradation, including bulk (elements inside the scaffold), surface (elements on the surface) degradation, as well as cell colonization on the scaffold by updating the elements’ mechanical properties.

A static stress analysis of the finite element model was conducted using ABAQUS/Standard 2022 (Simulia, Providence, RI, USA) to assess the mechanoregulation stimuli, which were then utilized as inputs for the agent-based model at each iteration.

To enhance the computational efficiency of the coupled simulation framework, the finite element analysis was performed once per three iterations (i.e., every three days). The temporal resolution was determined to have an insignificant effect on the overall accuracy of the model predictions.

#### 2.1.2. Agent-based cell and degradation model.

Agent-based modeling is a computational technique that represents individual agents, such as bone cells, as autonomous units governed by specific behavioural rules, allowing the simulation of complex and emergent dynamics within biological systems [54]. The full source code of the agent-based cell and degradation model is available on Zenodo (DOI: https://doi.org/10.5281/zenodo.18893712).

**Fig 4:**
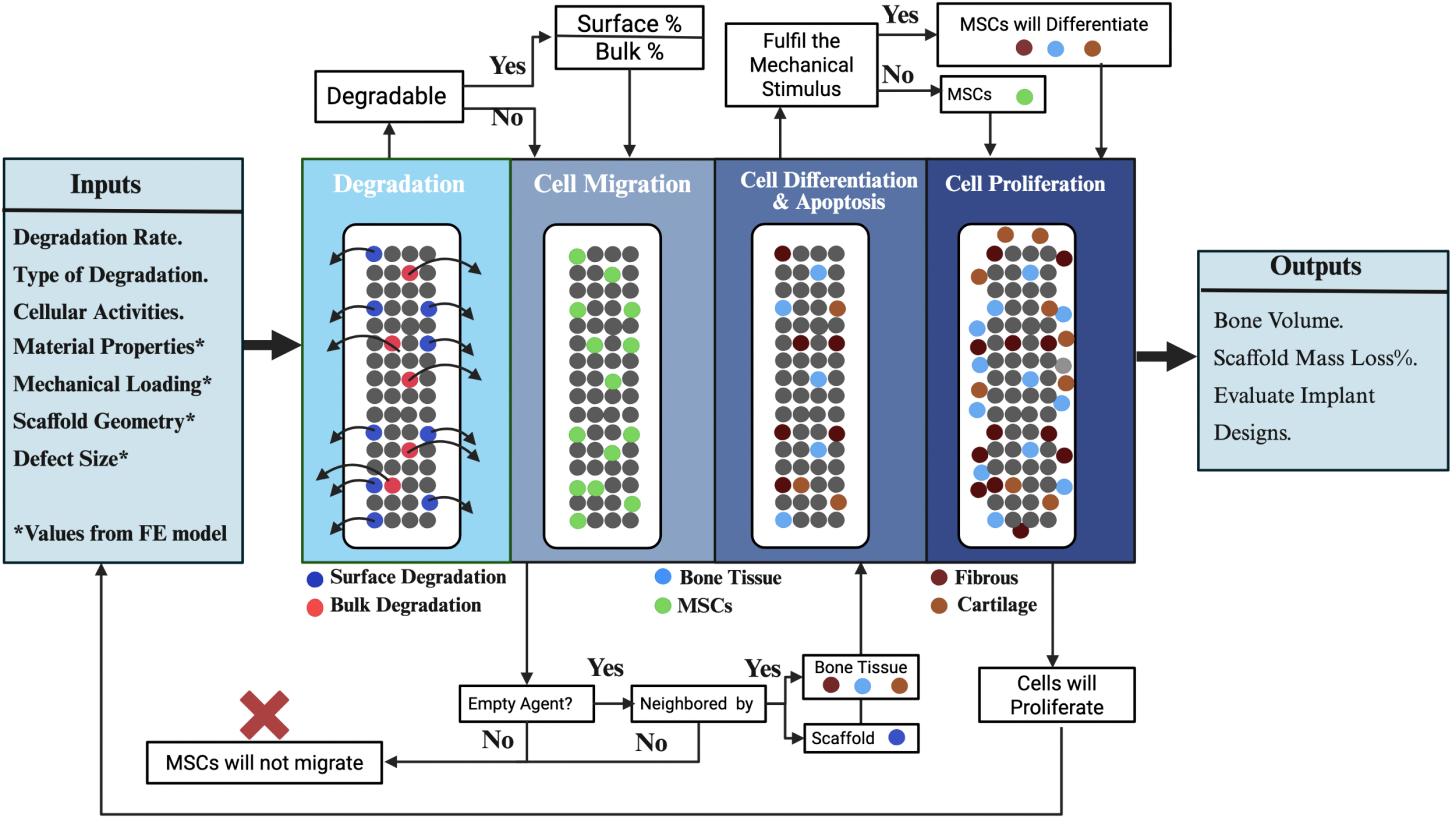
An agent-based cell and degradation model that uses stress based finite element analysis generated inputs to calculate scaffold degradation, tissue type, tissue volume and cell behavior.

An agent-based cell model was built based on our previous work [1], which simulated the actions and interactions of the different bone cells (i.e., MSCs, fibroblasts, chondrocytes, and osteoblasts). The actions of this model are cell seeding, migration, proliferation, differentiation, and apoptosis, as shown in Fig 4.

The agent-based degradation model simulates the surface and bulk degradations of the PLLA scaffold. The actions of this model are to allocate every agent in the scaffold and categorize it based on the equations (1–6), see Fig 6.

**Fig 5:**
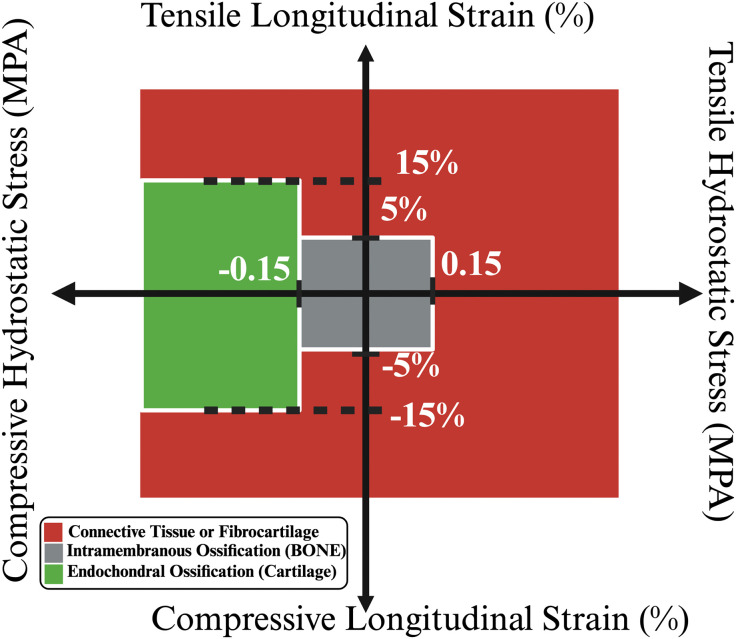
Mechanobiological model adapted from Claes et.al, (1999) [27] showing the relationship of mechanostimulus to tissue type adaptation.

**Fig 6:**
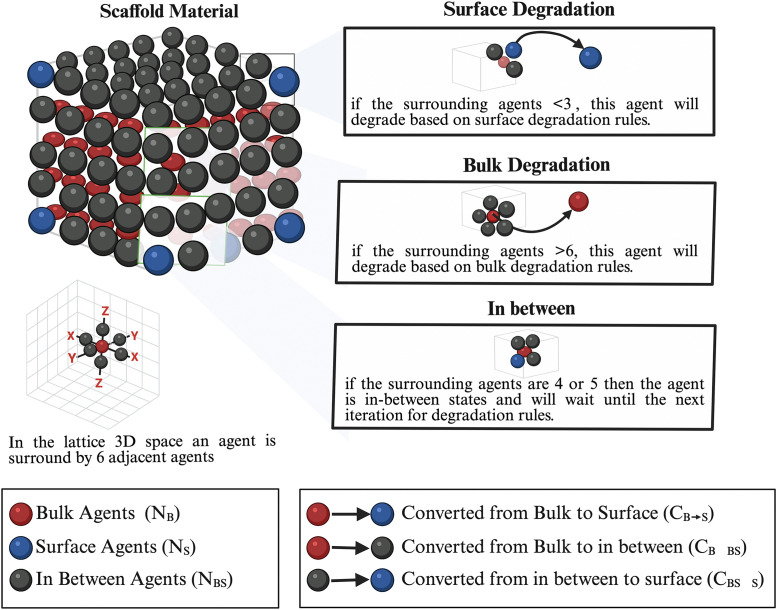
Agent-based schematic of scaffold degradation, implemented as a sub-module of the full agent-based model, where neighbour-count rules determine the degradation rules (surface: surrounded agents≤3; bulk: surrounded agents ≥6).

The agent-based model was developed utilizing the Python programming language (version 2.7.2). The outputs of strain and hydrostatic stress derived from the finite element model serve as inputs for the agent-based model, see Fig 4. A lattice framework was constructed with the same geometry as the callus volume with a lattice spacing of 50 µm. The lattice volume was set up with dimensions of 6 x 11 x 6 mm. The geometries of bones, callus, and scaffold are mapped into the lattice framework. The callus lattice is filled initially with agents representing granulation tissue, which then could change to different agent types (cell types), including MSCs, fibroblasts, chondrocytes, and osteoblasts, based on the different cell actions; see Fig 4. The scaffold lattice is filled with agents representing scaffold material, which then could change to granulation tissue based on the degradation process, see Fig 4.

The callus was initially composed of granulation tissue, and MSCs were derived from both the bone marrow cavity and the periosteum, as the periosteum is particularly rich in MSCs [55,56], 30% of the periosteum and marrow are seeded with MSCs as reported by Fan et. al [57]. A latency period of 7 days has been defined, which has been identified as the optimal duration followed by a significant reduction in cellular activity [58]. Previous *in silico* approaches utilized baseline rates of proliferation, apoptosis, differentiation, and the migration speed of MSCs, as illustrated in Table 2. Every agent type (cell type) in the callus lattice is surrounded by six agents representing either granulation, MSCs, fibroblasts, chondrocytes, osteoblasts or scaffold. MSCs agents migrate into these none cell occupied agents which are initially granulation tissue, which were set to migrate with a speed of 30µm/hour [59]. Cell proliferation action occurs randomly on one of the six neighbouring agents if they are not occupied by another cell type. MSCs differentiation action adheres to the mechanobiological principles outlined by Claes et al. [27] (see Fig 5), and the bone resorption zones [60].

**Table 2:** Cellular activities, including proliferation, apoptosis, differentiation, and migration rates per day [51,59].

	Proliferation rate per day		Apoptosis rate per day	Differentiation rate per day		Migration speed (µm/h)
	Before latency period	After latency period		Before latency period	After latency period	
Stem Cells	0.6	0.12	0.05	0.3	0.06	30
Fibroblasts	0.55	0.11	0.05	–	–	–
Chondrocytes	0.2	0.04	0.10	–	–	–
Osteoblasts	0.3	0.06	0.16	–	–	–

The degradation behaviour was implemented in this model by an iterative elimination of scaffold agents. The scaffold lattice agents are divided into three categories namely bulk agents (N_B_), surface agents (N_S_), and in between agents (N_BS_), see Fig 6. The degradation process occurs based on the eligibility of the scaffold agents within every category and based on the equations (1–5). The scaffold degraded agents then become part of the callus lattice and follow the agent-based cell model rules.

There are three scaffold agent specifications in the agent-based degradation model based on their positions within the scaffold lattice; see Fig 6:

**Surface agents (N**_**S**_) represent the agents located on the surface surrounded by three agents or less; these agents may increase or decrease and are only eliminated based on eligibility in every iteration.

**Bulk agents (N**_**B**_) represent the agents located only inside the scaffold lattice surrounded by 6 agents; these agents may become in between, or surface agents based on eligibility in every iteration.

**In between agents (N**_**B-S**_) represent all the agents located between the bulk agents and surface agents; these agents could change only to surface agents based on the new eligibility in every iteration.

**Converted surface agents from bulk agents (C**_**B->S**_) represent all agents converted from bulk to surface agents.

**Converted in between agents from bulk agents (C**_**B->B-S**_) represent all agents converted from bulk to in between agents.

**Converted surface agents from in between agents (C**_**B-S->S**_) represent all agents converted from bulk to in between agents.

Equation 1 describes the number of eliminated surface agents, E_S_,


$$\begin{array}{c} E_{S}(t)={D_{S}\cdot N}_{S}(t) \end{array}$$ (1)

Where D_S_ is the surface degradation rate, which is in our case 0.03 for PLLA within the body. Agents for elimination are then selected randomly until a total number of E_S_ agents are eliminated.

Equation 2 describes the number of surface agents in the next iteration (time step), $N_{S}(t+\text{1})$


$$\begin{array}{c} N_{S}(t+\text{1})=N_{S}(t)-E_{S}+C_{B\to S\;}(t)+C_{BS\to S\;}(t) \end{array}$$ (2)

Because of the elimination, new surface agents will become available.

Equation 3 describes the number of eliminated bulk agents, E_B,_


$$\begin{array}{c} E_{B}(t)={D_{B}\cdot N}_{B}(t) \end{array}$$ (3)

where, D_B_ degradation rate which is 0.00015 for PLLA within the body. Agents for elimination are then selected randomly until a total number of E_B_ agents are eliminated.

Equation 4 describes the number of bulk agents in the next iteration, N_B_ (t + 1)


$$\begin{array}{c} N_{B}(t+\text{1})=N_{B}(t)-\;E_{B}(t)-C_{B\to S}(t)-\;C_{B\to BS}(t) \end{array}$$ (4)

Because of the elimination, the new bulk agents could become N_BS_.

Equation 5 describes the number of new in between agents could become, N_BS_


$$\begin{array}{c} N_{BS}(t+\text{1})=N_{BS}(t)-C_{BS\to S}(t)+C_{B\to BS}(t) \end{array}$$ (5)

Equation 6 describes the total number of eliminated agents, E_Total_,


$$\begin{array}{c} E_{Total}(t)=E_{S}(t)+E_{B}(t) \end{array}$$ (6)

The agent-based cell and degradation model is updated every iteration, which is equivalent to one healing day, to conduct both biological actions and the degradation procedure. The changes based on the mechanical stress and strain are updated from the finite element model every three iterations to reduce the computational costs. All the data regarding agent type, position and material property are extracted and stored for every iteration. Which allows all the cells in the model to be tracked.

### 2.2. Analysis

#### 2.2.1. Validation case.

To validate the degradation model, *in vivo* experimental data from a prior study [61] served as a reference point. Three cylindrical scaffold geometries, each with dimensions of 5 mm diameter × 3 mm height, were created to emulate the internal architectures outlined in the study [61]. The scaffolds, designated as large pore size (PLLA-L), medium pore size (PLLA-M), and small pore size (PLLA-S), were designed with varying surface areas achieved through adjustments in filament and strut dimensions. Due to moulded manufacturing methods in the experimental study from the literature they refer to scaffold architecture being made from struts, whereas 3D printed scaffolds are made from filaments. In detail, the pore diameters and strut widths were 0.82 and 0.90 mm, 0.55 and 0.61 mm, and 0.28 and 0.42 mm for PLLA-L, PLLA-M and PLLA-S respectively.

**Fig 7:**
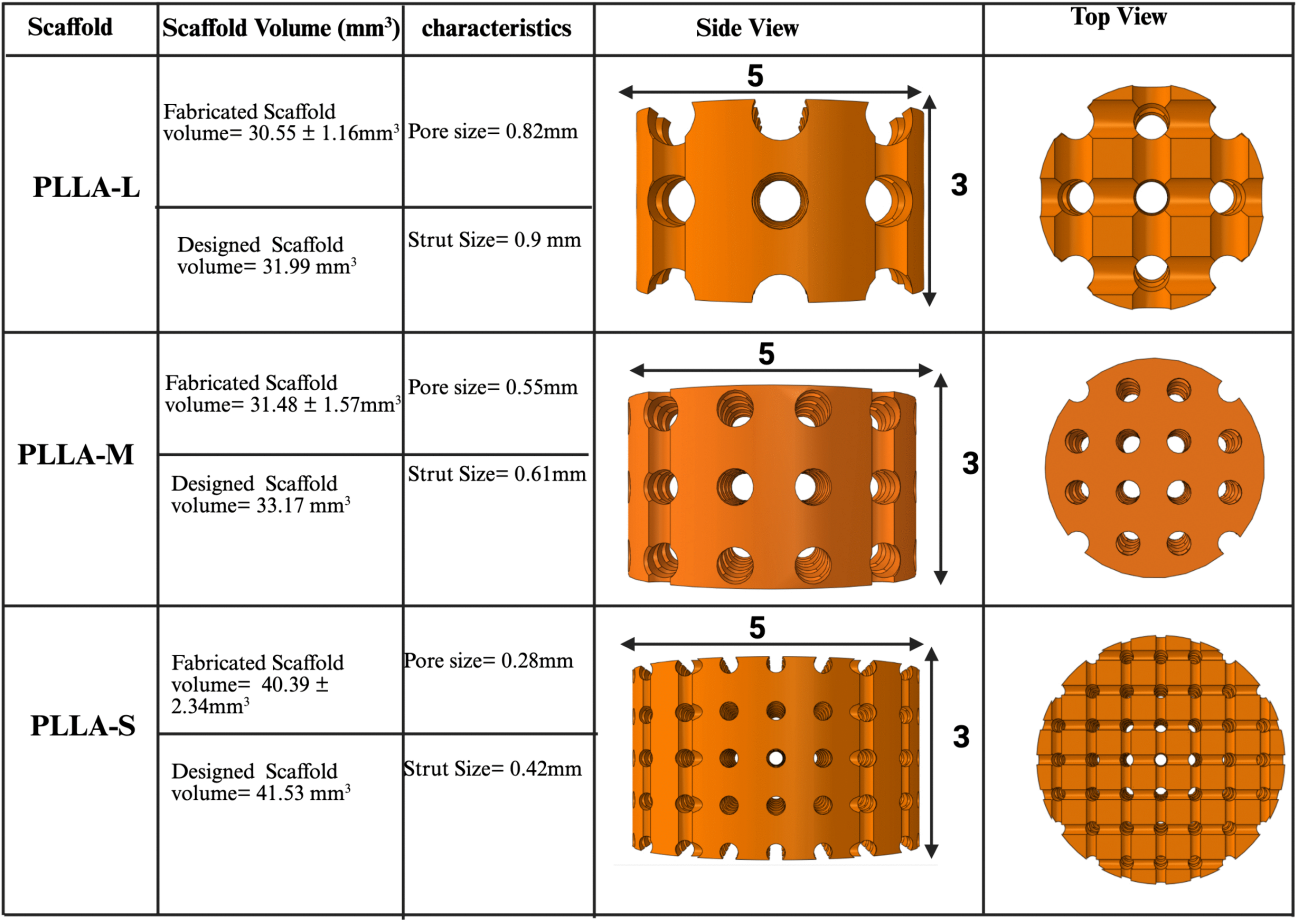
CAD model geometry for PLLA-L (large pore size), PLLA-M (medium pore size), and PLLA-S (small pore size) in relation to the scaffold fabricated throughout the experimental study [61].

The total volumes of the scaffolds were determined to be 31.99, 33.17, and 41.53 mm³ for PLLA-L, PLLA-M, and PLLA-S, respectively (see Fig 7). The parameters were subsequently employed to calibrate the computational model and equations used, guaranteeing that the simulated degradation rates accurately reflected those recorded in the *in vivo* experiments.

Scaffolds were subjected to degradation over a period of 21 weeks with no mechanical loading [61]. Mass loss calculated from the simulation was compared to the experimental degradation study [61]

#### 2.2.2. Case study: porosity & thickness effects on bone healing.

**Fig 8:**
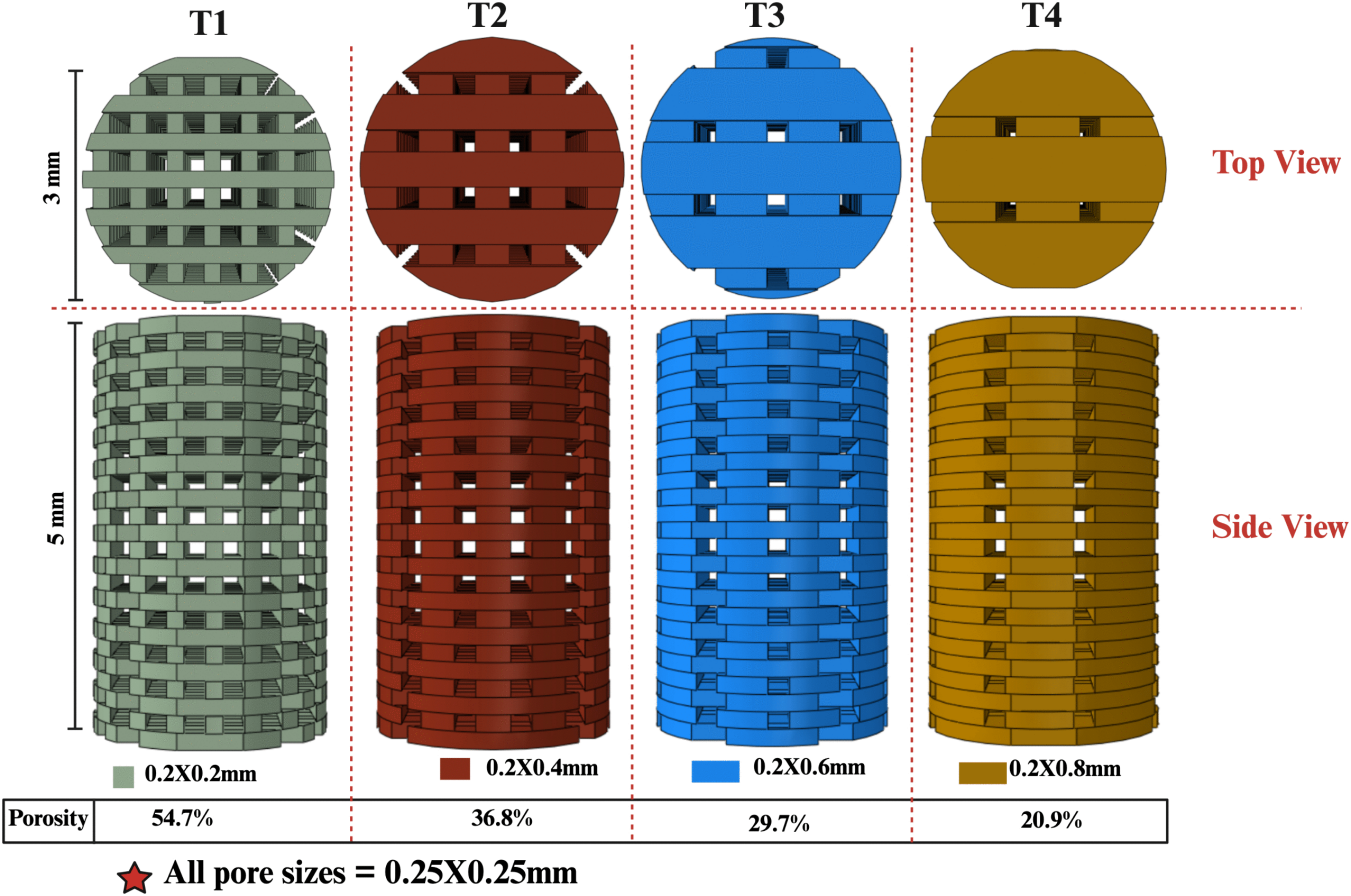
Scaffold designs featuring varying thicknesses and porosities utilized in the case study, illustrating both longitudinal and radial perspectives. T1 exhibits the thinnest scaffold with the greatest porosity. T2 exhibits scaffold designs characterized by increased thickness and reduced porosity, followed by T3 and T4, while porosity decreases progressively from T1 to T4.

Four case studies with different scaffold architectures, designated T1, T2, T3 and T4 (Fig 8), used the same bone defect conditions as described in sections 2.1.1 and 2.1.2 were outworked to investigate the effect of the scaffold architecture, specifically porosity and scaffold thickness, on bone formation and scaffold degradation. Scaffolds were developed using computer-aided design (CAD) to have a constant pore size to isolate the effects of filament dimensions. Filaments had a rectangular cross section measuring 0.2 × 0.2 mm, 0.2 × 0.4 mm, 0.2 × 0.6 mm, 0.2 × 0.8 mm, for T1, T2, T3 and T4, respectively; see Fig 8. This deliberate variation in filament thickness led to corresponding differences in the overall porosity of each scaffold model. By analyzing these constructs, we aim to elucidate how these geometric modifications affect the biological processes of bone tissue formation and the degradation kinetics of the scaffolds.

These PLLA scaffolds were subjected to mechanical loading as explained in sec 2.1.1 based on the experimental study [50], and based on the degradation rules as explained in sec 2.1.2, cellular activities (i.e., migration speed, proliferation, differentiation and apoptosis rates) were used from previous validated studies [51,59], as shown in Table 2. The mechanobiological model was run over 21 weeks.

## 3. Results

### 3.1. Validation case results (*in silico* outcomes compared to experimental results)

**Fig 9:**
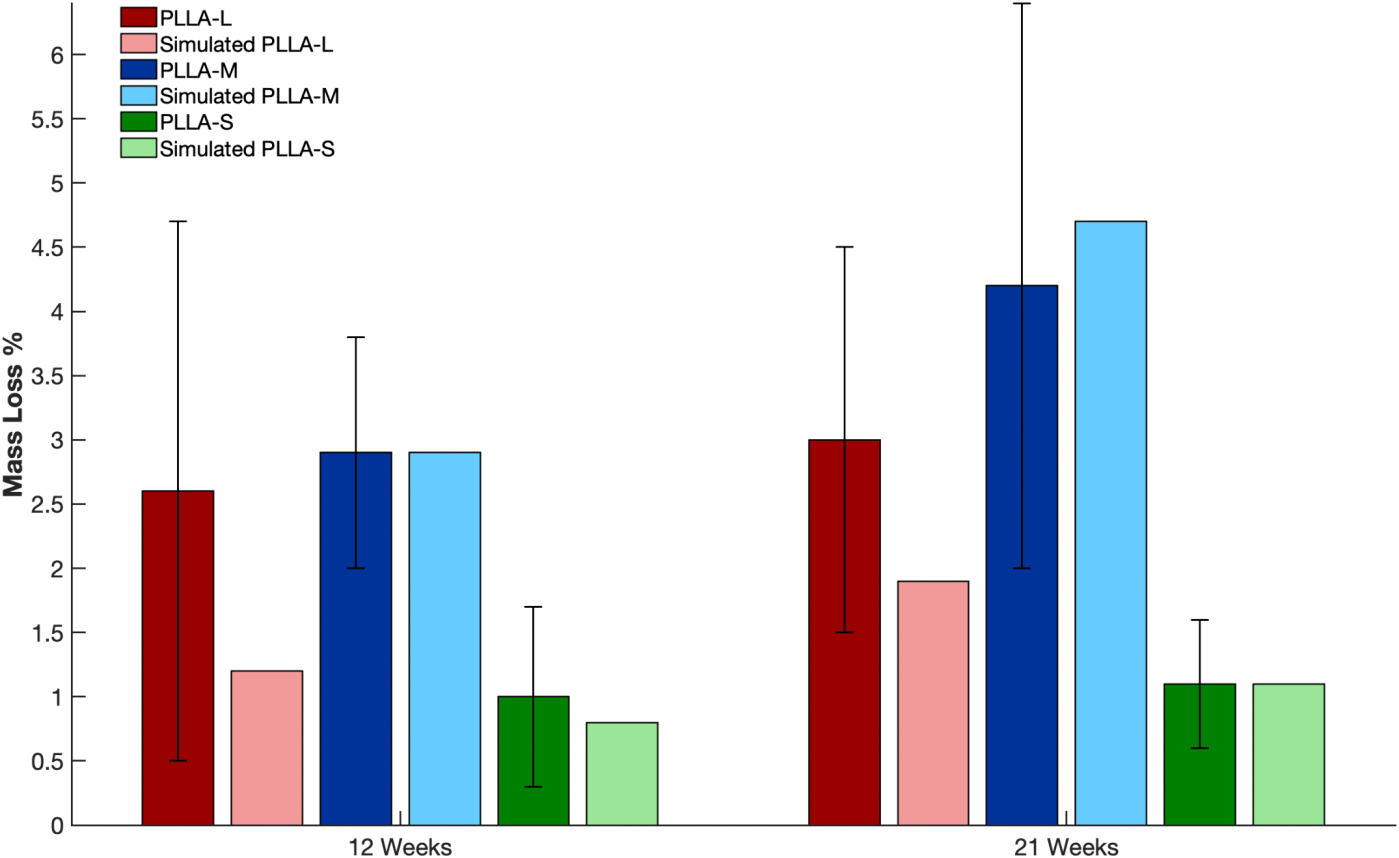
Graphs showing mass loss (%) for the three scaffolds at 12 and 21 weeks from the experimental and simulated results.

Fig 9 illustrates the experimentally observed mass loss percentages for the three scaffold types (PLLA-M, PLLA-L, and PLLA-S) in conjunction with the predictions from the computational model at 12, and 21 weeks. In both sets of results, PLLA-M consistently demonstrates the highest level of degradation, followed by PLLA-L and subsequently PLLA-S. The model predicts a mass loss of 2.9%, 1.2%, and 0.7% at the 12-week mark, respectively. At 21 weeks, PLLA-M shows the greatest mass loss at 4.9%, while PLLA-L and PLLA-S demonstrate mass loss of 1.9% and 1.1%, respectively. The model predictions are all within the standard deviation of the experimental results and the trends align with the experimental data. This confirms that PLLA-M degrades at the fastest rate, whereas PLLA-S shows the least impact under the tested conditions.

### 3.2. Case study results: the effect of the filament thickness architecture on bone formation and mass loss

#### 3.2.1. Mass loss.

**Fig 10:**
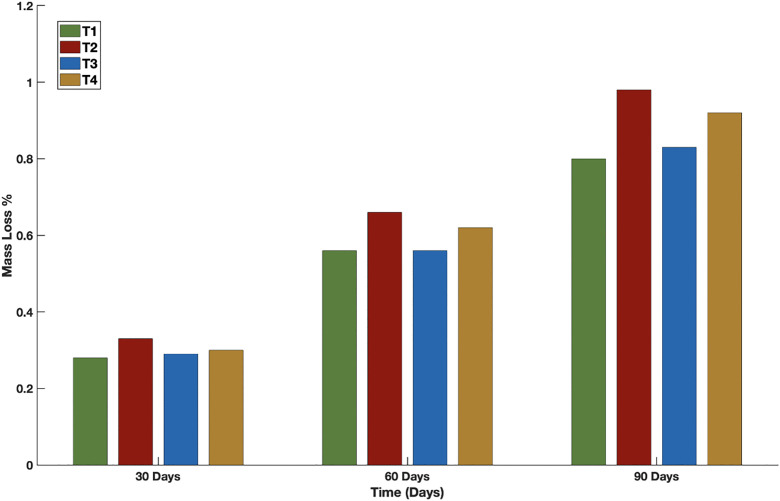
Mass loss through degradation from the case study of scaffolds T1, T2, T3 and T4 with different scaffold thicknesses and porosities at 30, 60 and 90 days.

All designs demonstrated a consistent increase in mass loss over time, with thicker filaments typically showing a higher degree of overall degradation. At 30 days, the anticipated mass loss for T2 was 0.34%, exceeding the figures of 0.29% for T1, and T3, and 0.32% for T4. At the 60-day, T2 exhibited the highest value at 0.67%, whereas the other designs varied between 0.57% and 0.63%. At the 90-day period, T2 achieved a mass loss of 0.97%, surpassing T1, T3, and T4 by margins of 0.17%, 0.14%, and 0.04%, respectively. The findings highlight the impact of filament thickness on degradation kinetics and reinforce the reliability of the computational model in differentiating between scaffold designs (Fig 10).

#### 3.2.2. Tissue formation.

**Fig 11:**
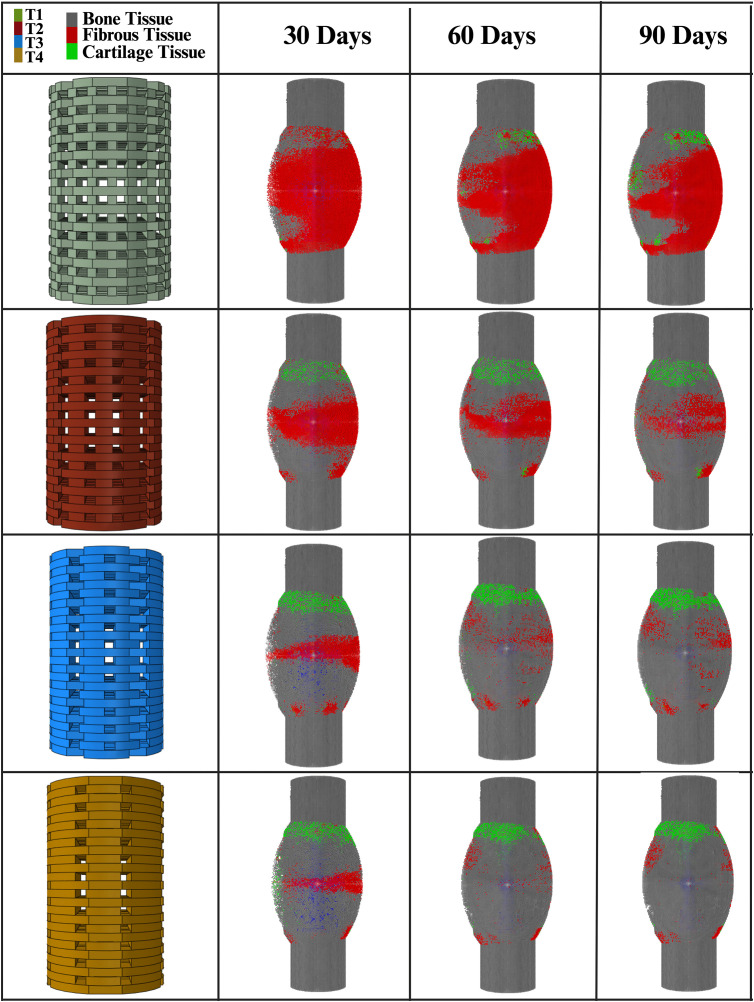
Graphical image of the tissue formation (fibrous, cartilage and bone) predicted for the four scaffold architectures (T1, T2, T3 and T4).

**Fig 12:**
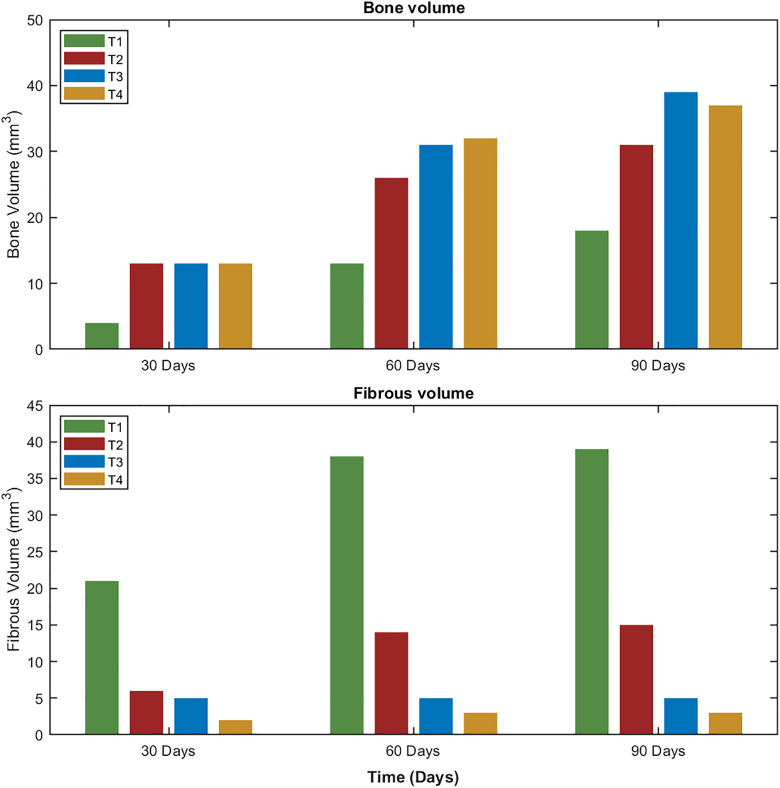
Bone and fibrous tissue volume for the four scaffold architectures (T1, T2, T3 and T4) at 30, 60 and 90 days.

Fig 11 presents the predicted changes in bone volume over a 90-day period for the four different scaffold architectures (T1, T2, T3, and T4). The results demonstrate that scaffold architecture plays a critical role in bone formation, with thicker scaffolds promoting greater bone regeneration, see Fig 12. Bone formation increased progressively over time in all cases, with T3 exhibiting the highest bone volume at each time point, followed closely by T2 and T4, while T1 displayed the lowest bone formation throughout the study period. At 90 days, T3 produced the highest bone volume, surpassing T4, T2, and T1. These findings suggest that scaffold thickness and porosity significantly influence osteogenesis, highlighting the potential of optimized scaffold designs to enhance bone regeneration.

Fig 12 depicts the progression of fibrous tissue formation from 30 to 90 days for the four scaffold architectures (T1, T2, T3, and T4). Notably, T1 exhibited the highest level of fibrous formation, surpassing T2, T3, and T4 by 24.8, 34.3, and 36.1 mm³, respectively. These findings highlight the influence of scaffold architecture on cartilage regeneration, with T1 demonstrating superior capacity for supporting new fibrous tissue growth.

#### 3.2.3. Scaffold thickness & porosity effects on MSCs activity and osteogenic outcomes.

**Fig 13:**
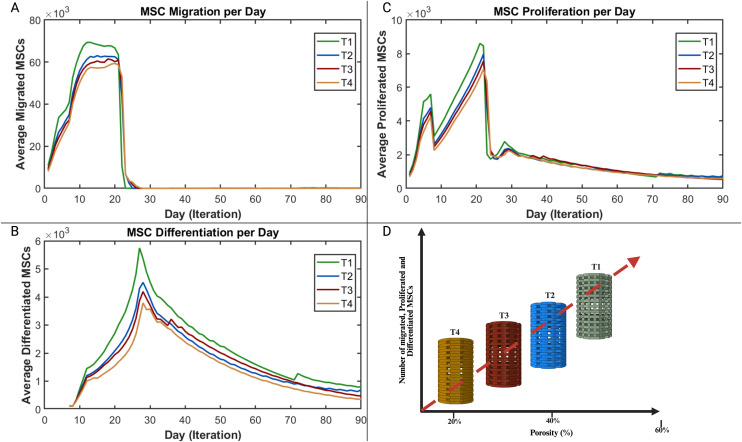
Cellular dynamics of MSCs within scaffolds of varying thicknesses and porosities. **(A)** Migration profiles showing the number of migrated MSCs per day. **(B)** Differentiation trajectories indicating differentiated MSCs per day. **(C)** Proliferation patterns demonstrating proliferated MSCs per day. **(D)** Schematic summary showing the general trend of the MSCs dynamics for the different activities.

**Fig 14:**
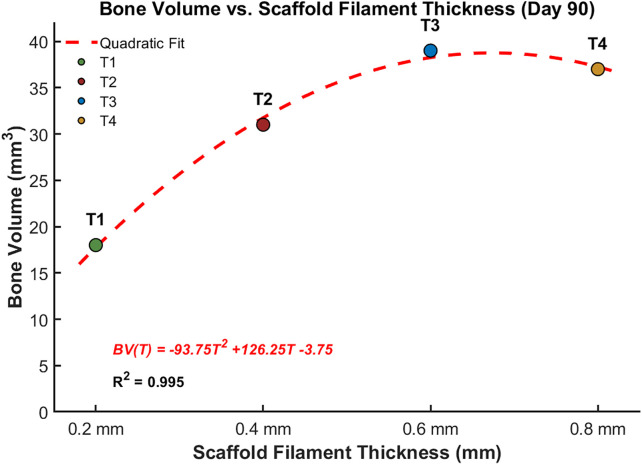
Relationship of the predicted regenerated bone volume after 90 days and different scaffold thicknesses.

The results revealed a distinct influence of scaffold filament thickness and corresponding porosity on the temporal dynamics of mesenchymal stem cell (MSC) behavior and subsequent osteogenic outcomes. As shown in Fig 13(A–D), scaffolds with thinner filaments and higher porosity (T1) exhibited the highest levels of MSC migration, proliferation, and differentiation during the early stages (approximately days 10–25), followed by T2, T3, and T4. This indicates that increased porosity facilitates greater cellular infiltration and expansion due to enhanced nutrient diffusion and available surface area for cell attachment. However, the pattern of osteogenic differentiation and bone tissue formation, illustrated in Fig 14, followed an opposite trend. The scaffold with intermediate filament thickness (T3) demonstrated the highest osteoblast activity and bone formation potential, followed by T4, T2, and T1. This parabolic relationship suggests that while highly porous scaffolds promote rapid initial cellular activity, an intermediate pore architecture (T3) provides the optimal balance between mechanical stability and biological accessibility necessary for effective osteogenesis. Excessive porosity, as in T1, may reduce mechanical support and limit the maturation of osteogenic tissue despite the initial increase in MSC activity.

From our model’s simulation results the bone volume, BV, can be related to the scaffold filament thickness, T, by equation 7, which is derived by least squares curve fitting a quadratic fit.


$$\begin{array}{c} BV(T)=-\text{9}\text{3}.\text{7}\text{5}\;T^{\text{2}}+\text{1}\text{2}\text{6}.\text{2}\text{5}\;T-\text{3}.\text{7}\text{5}\; \end{array}$$ (7)

This shows that scaffold filament thickness, T, has a local maximum or optimum value to maximize new bone volume, BV(T), formation.

## 4. Discussion

In this study, we developed a mechanobiological model that combines an agent-based cell model with an agent-based degradation model coupled with a finite element model of the stress environment to simulate and predict the dynamic interplay between scaffold degradation and bone regeneration. To the author's knowledge, this approach represents the first attempt to couple cellular activities with the effect of scaffold degradation, including bulk and surface degradation. Our model was validated against experimental data from three scaffold architectures implanted *in vivo* [61] and showed good agreement with the *in vivo* results within the standard deviation of the reported data. Our model was used to systematically vary scaffold filament thickness and porosity to reveal that scaffold architecture plays a critical role in directing degradation rate, cellular behaviour and tissue regeneration outcomes.

In this study, we examined the degradation behaviour of scaffolds T1, T2, T3, and T4, which were engineered with consistent pore sizes yet differing filament thicknesses, leading to variations in porosity. T1, characterized by its higher porosity (~54.7%) resulting from thinner filaments, demonstrated the least mass loss over a 90-day period. In contrast, T2, characterized by its intermediate filament thickness and porosity, exhibited the greatest mass loss. This indicates that achieving an ideal equilibrium between filament thickness and porosity may improve degradation rates. This is because the design of the scaffold encourages the highest number of the scaffold agents to be eligible for both bulk (D_b_) and surface agent (D_s_) categories, as shown in Fig 4. These outcomes align with previous experimental investigations which showed that scaffolds exhibiting moderate porosity and suitable filament dimensions can promote effective degradation [62]. In contrast, T3 and T4, which are defined by their thicker filaments and lower porosity (~29.7% and ~20.9%, respectively), demonstrated decreased degradation rates. This trend aligns with findings indicating that reduced porosity can hinder fluid diffusion, consequently slowing down the degradation process. The findings emphasize the essential influence of filament thickness and porosity on scaffold degradation, stressing the importance of a well-considered design to attain optimal degradation rates for applications in tissue engineering.

Our findings indicate that scaffolds exhibiting greater porosity markedly improve the migration and proliferation of MSCs. Previous studies highlight that pore interconnectivity is significant for nutrient diffusion and cell infiltration. [63,64]. This advantageous microenvironment promotes early tissue regeneration by guaranteeing adequate nutrient transport and strong cell colonization, see Fig 13.

Several mechanobiological models represent bone regeneration by assigning distinct material properties to immature, intermediate, and mature bone phases in order to approximate progressive mineralization and stiffness evolution [32,65,66]. In contrast, the present study adopts a stimulus-based mechanoregulation framework in which the local mechanical environment governs differentiation into fibrous tissue, cartilage, or bone. Once bone tissue is formed, it is represented as a single material phase rather than subdivided into multiple maturation states. Our approach enables investigation of architecture-dependent and degradation-driven effects within a consistent mechanobiological framework while preserving the fundamental mechanically regulated differentiation patterns observed during new bone tissue formation. But it should be noted that future work could build on this approach by also including distinct phases of bone as well.

Scaffold architecture, specifically filament thickness and porosity, plays a pivotal role in regulating both degradation behaviour and bone regeneration. Our findings are consistent with prior experimental studies demonstrating that highly porous scaffolds composed of thin filaments promote increased surface erosion due to their elevated surface-to-volume ratio. For instance, Gleadall et al. (2014) reported that porous PCL scaffolds with 90% porosity showed significantly faster degradation than those with lower porosity [67]. Similarly, Karande et al. (2004) observed that scaffold design greatly influenced fluid transport and thus degradation kinetics [68]. In the context of cellular behaviour, our observation that T1 scaffolds facilitated greater MSC migration and proliferation aligns with the work of O’Brien (2011), who emphasized the importance of pore interconnectivity in enhancing nutrient diffusion and cell ingress [69]. However, the reduced mechanical stiffness of T1 likely contributed to fibrous tissue formation rather than osteogenesis, which is consistent with the mechanobiological model proposed by Claes and Heigele (1999) [27], where inadequate mechanical stimulus steers MSCs away from the osteogenic lineage. Conversely, our T4 scaffold configuration, featuring thicker filaments and lower porosity, demonstrated slower degradation kinetics and promoted osteogenic differentiation, though at the cost of reduced cell infiltration, an outcome corroborated by studies from Karageorgiou and Kaplan (2005), who found that low-porosity scaffolds prevent cellular infiltration and matrix deposition [63]. The intermediate configurations, particularly T3, balanced mechanical support with adequate porosity, producing the most favorable bone regeneration outcomes. These results emphasize the importance of optimizing scaffold architectural features to match biological accessibility with mechanical integrity in order to achieve both effective degradation profiles and improved osteogenesis.

The structure and filament dimensions of the scaffolds significantly affect the mechanical stability crucial for bone formation. Enhanced mechanical support was typically observed with thicker scaffold filaments, resulting in greater bone formation. Nonetheless, our investigation uncovered a complex interplay between filament thickness and tissue outcomes. While the thickest configuration (T4) demonstrated improved mechanical properties, it did not result in the greatest bone volume. The T3 configuration demonstrated better performance compared to T4, as illustrated in Fig 14. This is likely attributed to nutrient diffusion limitations inherent in the denser T4 architecture, which hindered cell migration and proliferation, as shown in Fig 13.

The scaffold characterized by the thinnest filament thickness (T1) demonstrated the most significant levels of MSC migration and proliferation. Despite this cellular advantage, T1 resulted in the lowest bone formation and instead led to an increase in fibrous tissue, as illustrated in Fig 12. This contradiction can be elucidated by the insufficient mechanical support it receives. As outlined in the mechanobiological framework proposed by Claes and Heigele (1999) [27], see Fig 4, an ideal mechanical stimulus is essential for guiding MSC differentiation into osteoblasts. The inadequate mechanical environment in the case of T1 probably promoted differentiation into fibroblasts, resulting in increased fibrous tissue formation instead of bone deposition, as illustrated in Fig 12 and 14.

A key advancement of our research is the integration of an agent-based identification strategy into our comprehensive model. This approach enables the reclassification of degraded scaffold agents as integral to the developing bone, with their material characteristics adapting in response to local cellular interactions. This method addresses the constraints linked to a fixed callus area and provides a more realistic representation of the tissue-scaffold interface.

The integrated model we developed, which combines mechano-stimuli with degradation kinetics, holds great potential for enhancing scaffold design in bone tissue engineering. The model's inherent flexibility enables calibration against diverse material degradation rates, broadening its applicability beyond PLLA to include other biodegradable materials. This capability not only enhances the accuracy of predictive simulations but also allows for the refinement of scaffold architectures that need experimental validation, thus minimizing the total number of *in vivo* and *in vitro* studies required, following the three Rs approach (replacement, reduction and refinement). As a result, this method has the potential to enhance the efficiency of the scaffold optimization process, reducing both time and expenses. Furthermore, by clarifying the relationship between scaffold degradation and bone regeneration, our model offers enhanced understanding of the mechanobiological processes that facilitate tissue repair. The findings can guide the thoughtful design of scaffolds that strike an ideal balance between mechanical support and biological functionality, thereby speeding up the process of bringing engineered scaffolds into clinical use.

Biomechanical loading was applied in a simplified static form based on peak rat femoral gait values to approximate physiological conditions [50]. Although bone healing is inherently dynamic and cyclic, static loading is commonly employed in mechanobiological models [1,36,45] as a computationally efficient representation of the dominant mechanical stimulus governing tissue differentiation. The model operates iteratively, with each step representing one day of healing during which scaffold degradation and tissue stiffness are updated, thereby altering the mechanical stimulus field. Micromotion is not explicitly imposed but emerges implicitly from the evolving strain distribution within the finite element model. Fixation stiffness and boundary conditions were kept constant to provide a controlled framework for evaluating architecture-dependent effects, representing a simplified treatment of the screw–bone interface behaviour.

Despite the advancements presented herein, several limitations remain. In our previous work, a predefined callus shape was generated *in silico* and filled with granulation tissue, although *in vivo* callus formation was not observed [1,33,34,36,44,70]. In the current study, this limitation was partially mitigated by reclassifying degraded scaffold elements from the Abaqus simulation as agents of the agent-based cell and degradation model, thereby incorporating them into the bone tissue formation process. Another limitation is that no revascularization process of the defect was included in this simulation, despite its recognized importance in large bone defect regeneration [1,32,33,36]. In the present framework, this was mitigated by allowing bone formation only in regions that satisfy appropriate biological and mechanical conditions (the callus), but this is an oversimplification of in vivo behaviour. Future work should include revascularization processes within the mechanobiological model.

Our work attempts to improve bone tissue scaffold degradation modelling by including both surface and bulk degradation processes. This is important as our work is focused on the influence of shape, rather than material, where bulk and surface degradation will significantly influence the changing geometry of the scaffold which will improve the modelling of both the mechanical and biological processes. However, degradation is a complex process, and our model uses fixed rates of surface and bulk degradation. This means that we have not included the effects of localized pH, fluid flow, crystallinity and autocatalysis which are important chemical and environmental factors. This is a limitation of the model and future work should build on these areas. But a sensitivity analysis was performed on various degradation rates between ten times slower and ten times faster than the case used, and new tissue volume was only changed by 1 mm³ (see Supplementary Information).

A partial validation of the model is focused on degradation behaviour through comparison with experimental mass loss data under loading-free conditions. The mechanical and biological predictions combined with degradation were not directly validated in this study. However, the mechanical and biological model without degradation was validated based on bone tissue formation in our previous work [1]. Future studies could use additional histological analysis and micro-CT imaging to fully validate mass loss and tissue formation in the same data set.

The results emphasize the intricate relationships among scaffold degradation, porosity, filament thickness, mechanical stability, and cellular activity in the context of bone tissue engineering. The emphasis is placed on the necessity for a scaffold design that effectively balances mechanical support with biological functionality. Future studies will concentrate on enhancing these computational models and confirming their predictions with additional experimental data, ultimately promoting the advancement of optimized scaffold designs for clinical applications in bone regeneration.

## 5. Conclusions

Our study created an innovative mechanobiological model that combines an agent-based cell and degradation model coupled with a finite element model of the stress environment to simulate and predict the dynamic interplay between scaffold degradation and bone regeneration. This model has undergone validation using *in vivo* experimental data from PLLA scaffolds with three distinct architectures (PLLA-L, PLLA-M, and PLLA-S), demonstrating its ability to reliably predict degradation patterns and tissue formation results.

We investigated the effect of scaffold architecture, specifically porosity and filament thickness, on bone formation and scaffold degradation kinetics. The results indicate that increased scaffold porosity significantly enhances the migration and proliferation of mesenchymal stem cells. In contrast, scaffolds featuring thicker filaments, although providing greater mechanical support, could impede nutrient diffusion and adversely affect bone formation.

Furthermore, the case studies demonstrate that variations in scaffold thickness and porosity significantly influence degradation rates and the ensuing process of bone regeneration. The configuration featuring the thinnest filaments (T1) promotes significant cell migration; however, it results in increased fibrous tissue formation due to insufficient mechanical stimulation, consistent with the mechanobiological findings of Claes and Heigele (1999) [27].

In future studies our model could be adjusted for various material degradation rates, which could streamline the experimental design process and enables a more focused strategy for scaffold optimization and full exploration of scaffold architecture (implant shape design space). Our investigation highlights the crucial role of scaffold geometry in the field of bone tissue engineering and provides a strong predictive framework for the design and enhancement of biodegradable implants.

## Supporting information

S1 Fig Agent-based model workflow diagram. (TIF)

S1 Table Agent-based models reporting terminology. (DOCX)

S2 Table Sensitivity analysis of total tissue formation at Day 90 under varying scaffold degradation rates (slower degradation by 10x–faster degradation rate by 10x compared to the baseline degradation case (T1)). (DOCX)

S3 Table Sensitivity analysis of the total number of cells and osteoblast cells at Day 90 for the baseline degradation case (T1) applied either to bulk degradation only or to surface degradation only. (DOCX)
