# Supplementary material for: Tissue scaffold architecture affects implant degradation and bone tissue regeneration: A novel in silico mechanobiological model analysing cell behavior, mechanical stress and degradation kinematics
Source: PLoS One. 2026 May 28;21(5):e0349708. doi: 10.1371/journal.pone.0349708 (PMC13218534; doi:10.1371/journal.pone.0349708)
Supplement: S1 Table — (DOCX) [file pone.0349708.s002.docx]

**S1 Table.** Agent-based models reporting terminology

| **Term** | **Definition** |
| --- | --- |
| Lattice global volume | The volume in which physical parts are mapped imported into |
| Lattice spacing | The agent size |
| Mapping | Tracking physical parts (elements and nodes in the FE input file) to convert it into agents in the lattice global volume. |
| Agent | The unit cell of all the lattice global volume. |
| Agent type | Cell, tissue or medical implant… etc. |
| Agent change relationship | The rules that govern how agents should behave in every iteration. |
| Iteration | The execution of the code, based on the rules could equal any actual duration (i.e., an hour, a day, a week… etc). |
