# Supplementary material for: Tissue scaffold architecture affects implant degradation and bone tissue regeneration: A novel in silico mechanobiological model analysing cell behavior, mechanical stress and degradation kinematics
Source: PLoS One. 2026 May 28;21(5):e0349708. doi: 10.1371/journal.pone.0349708 (PMC13218534; doi:10.1371/journal.pone.0349708)
Supplement: S2 Table — (DOCX) [file pone.0349708.s003.docx]

**S2 Table.** Sensitivity analysis of total tissue formation at Day 90 under varying scaffold degradation rates (slower degradation by 10x–faster degradation rate by 10x compared to the baseline degradation case (T1)).

| **Degradation Rate**  **(combined surface and bulk)** | **Total Cells**  **(number of cells)** | **Tissue Volume**  **(mm³)** |
| --- | --- | --- |
| Reduced by10x | 519,461 | 64.9 |
| Reduced by 4x | 519,542 | 64.9 |
| **Normal** | 519,726 | 65.0 |
| Increased by 4x | 519,871 | 65.0 |
| Increased by 10x | 520,256 | 65.0 |
