# Supplementary material for: Tissue scaffold architecture affects implant degradation and bone tissue regeneration: A novel in silico mechanobiological model analysing cell behavior, mechanical stress and degradation kinematics
Source: PLoS One. 2026 May 28;21(5):e0349708. doi: 10.1371/journal.pone.0349708 (PMC13218534; doi:10.1371/journal.pone.0349708)
Supplement: S3 Table — (DOCX) [file pone.0349708.s004.docx]

**S3 Table.** Sensitivity analysis of the total number of cells and osteoblast cells at Day 90 for the baseline degradation case (T1) applied either to bulk degradation only or to surface degradation only.

| Increased by 10X | Total Cells | Osteoblast Cells |
| --- | --- | --- |
| Only Bulk | 518,869 | 133,064 |
| Baseline case | 519,726 | 139,843 |
| Only Surface | 519,790 | 144,888 |
